# Assessment of CD200R Activation in Combination with Doxycycline in a Model of Melioidosis

**DOI:** 10.1128/spectrum.04016-22

**Published:** 2023-05-18

**Authors:** R. E. Thom, E. D. Williamson, J. Casulli, W. A. Butcher, G. Burgess, T. R. Laws, P. Huxley, R. Ashfield, M. A. Travis, R. V. D’Elia

**Affiliations:** a CBR Division Defence Science and Technology Laboratory Porton Down, Salisbury, United Kingdom; b Lydia Becker Institute for Immunology and Inflammation, Wellcome Trust Centre for Cell-Matrix Research, Faculty of Biology, Medicine and Health, Manchester Academic Health Sciences Centre, University of Manchester, Manchester, United Kingdom; c Ducentis BioTherapeutics Ltd., Oxford, Oxfordshire, United Kingdom; d Strathclyde Institute of Pharmacy & Biomedical Sciences, University of Strathclyde, Glasgow, United Kingdom; Hartford Hospital

## Abstract

Antimicrobial resistance continues to be a global issue. Pathogens, such as Burkholderia pseudomallei, have evolved mechanisms to efflux certain antibiotics and manipulate the host response. New treatment strategies are therefore required, such as a layered defense approach. Here, we demonstrate, using biosafety level 2 (BSL-2) and BSL-3 *in vivo* murine models, that combining the antibiotic doxycycline with an immunomodulatory drug that targets the CD200 axis is superior to antibiotic treatment in combination with an isotype control. CD200-Fc treatment alone significantly reduces bacterial burden in lung tissue in both the BSL-2 and BSL-3 models. When CD200-Fc treatment is combined with doxycycline to treat the acute BSL-3 model of melioidosis, there is a 50% increase in survival compared with relevant controls. This benefit is not due to increasing the area under the concentration-time curve (AUC) of the antibiotic, suggesting the immunomodulatory nature of CD200-Fc treatment is playing an important role by potentially controlling the overactive immune response seen with many lethal bacterial infections.

**IMPORTANCE** Traditional treatments for infectious disease have focused on the use of antimicrobial compounds (e.g. antibiotics) that target the infecting organism. However, timely diagnosis and administration of antibiotics remain crucial to ensure efficacy of these treatments especially for the highly virulent biothreat organisms. The need for early antibiotic treatment, combined with the increasing emergence of antibiotic resistant bacteria, means that new therapeutic strategies are required for organisms that cause rapid, acute infections. Here, we show that a layered defense approach, where an immunomodulatory compound is combined with an antibiotic, is better than an antibiotic combined with a relevant isotype control following infection with the biothreat agent *Burkholderia pseudomallei*. This approach has the potential to be truly broad spectrum and since the strategy includes manipulation of the host response it's application could be used in the treatment of a wide range of diseases.

## OBSERVATION

Burkholderia pseudomallei is the causative agent of melioidosis. The bacterium has evolved mechanisms to evade innate host defenses and to persist (1), making it highly virulent if left untreated (2, 3). These evasion mechanisms significantly contribute to the reduced efficacy of traditional medical countermeasures, such as antibiotics, in treating melioidosis (4).

A range of host natural regulators which typically maintain immune homeostasis when administered as immunomodulatory compounds have demonstrated therapeutic benefits for the treatment of infectious disease (5–9). The type 1 transmembrane glycoprotein CD200 receptor (CD200R) is present on cells of the myeloid lineage (10) and is highly expressed on alveolar macrophages and neutrophils (5). We have shown that mice lacking CD200R displayed increased infectious burden after Francisella tularensis LVS infection which was due to dysfunctional neutrophil activation and a reduction in reactive oxygen species (6). Targeting this pathway to induce immunomodulatory effects, as well as antimicrobial effector functions, could benefit the field of infectious diseases, specifically in the treatment of patients with melioidosis.

Despite Burkholderia pseudomallei being endemic in countries throughout Asia (11), there remains a requirement for animal models to test novel therapeutic strategies before transitioning to human clinical trials. The CBR Division Defence Science and Technology Laboratory (Dstl) and others have demonstrated the utility of murine models to mimic the different forms of the disease. As with all *in vivo* model studies, there are limitations to the translatable nature of the data generated, but murine *Burkholderia* vaccine data have been used recently to support up-and-coming vaccine trial studies (12, 13).

**FIG 1:**
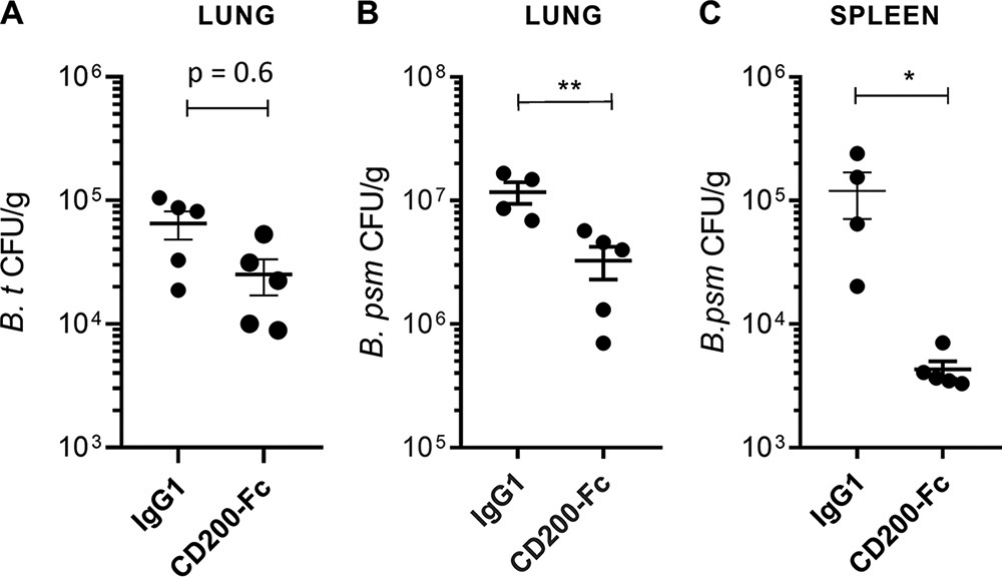
CD200-Fc sole therapy for the treatment of *Burkholderia* species. (A) C57BL/6 mice (*n* = 10 for each treatment group) were challenged with 5,000 CFU B. thailandensis (B.t) via the intranasal (i.n.) route and then treated with either CD200-Fc or isotype control administered via the i.n. route at 6 h post-infection. (B and C) BALB/c mice (*n* = 5 for each treatment group) were challenged with 50 CFU (12.5 LD_50_) B. pseudomallei via the aerosol route and then treated with CD200-Fc or isotype control administered via the i.p. route at 1 day post-infection. Bacterial burden within the lung (A and B) and spleen (C) was determined at day 3 post-infection and defined as CFU/gram of tissue (CFU/g) relative to the average IgG1 control. The data were analyzed using Student’s *t* test; *, *P* < 0.05; **, *P* < 0.01. All data represent mean ± SEM.

BSL-2 *in vivo* studies were conducted with groups of C57BL/6 mice infected via the intranasal (i.n.) route with 5,000 CFU Burkholderia thailandensis (E264, ATCC number 700388). At 6 h post-infection, mice were treated either with CD200-Fc (research grade; R&D Systems) or IgG isotype control (i.n. administration at 1.25 mg/kg of body weight) and monitored for 3 days. At the end of the study, mice were culled and lung tissue was removed for bacterial enumeration. In the lung, following treatment with CD200-Fc, there was a lower bacterial load than mice treated with IgG; however, this difference did not reach statistical significance (Fig. 1A).

This initial BSL-2 model suggested that CD200-Fc warranted further exploration for the treatment of the BSL-3 pathogen B. pseudomallei. BALB/c mice were challenged with 50 CFU (12.5 50% lethal dose [LD_50_]) B. pseudomallei (K96243) via the aerosol route (using the Biaera aerosol management platform) and treated at 1 day post-infection with CD200-Fc or IgG control via the intraperitoneal (i.p.) route. Culls were performed at day 3 post-infection, and organ tissues were collected. Bacterial burden in the lung and spleen was significantly reduced following CD200-Fc treatment compared with that of the control (Fig. 1B and C).

The treatment of melioidosis currently requires antibiotic treatment (14, 15), which typically involves a combination of antibiotics (16, 17). Reliance on antibiotics increases the likelihood of antimicrobial resistance, and the potential use of B. pseudomallei as a biological threat agent necessitates the requirement of novel therapies for acute infection. Thus, a combination treatment strategy, to include CD200-Fc in addition to antibiotics, could have further therapeutic benefits. This combination therapy could protect against acute infection, by targeting persistent bacterial phenotypes and by protecting against reinfection through supporting the local host environment.

Doxycycline was used in these studies, which although it has been recommended for eradication in the past (16), it has now been shown to be ineffective alone to clear melioidosis (14). Using a suboptimal antibiotic with CD200-Fc as a combination therapy will help determine the therapeutic benefit of the immunomodulator. The effectiveness of CD200-Fc in combination with doxycycline was tested in mice following infection with B. pseudomallei up to 35 days. This study was first carried out using CD200-Fc obtained from R&D Systems. We repeated this study to confirm findings using a second source of murine CD200-Fc provided by Ducentis, which was modified to allow high-affinity binding to the CD200 receptor. The high-affinity CD200-Fc (haCD200-Fc) compound has been used by the company as a stepping-stone to clinical trials with a human analogue.

**FIG 2:**
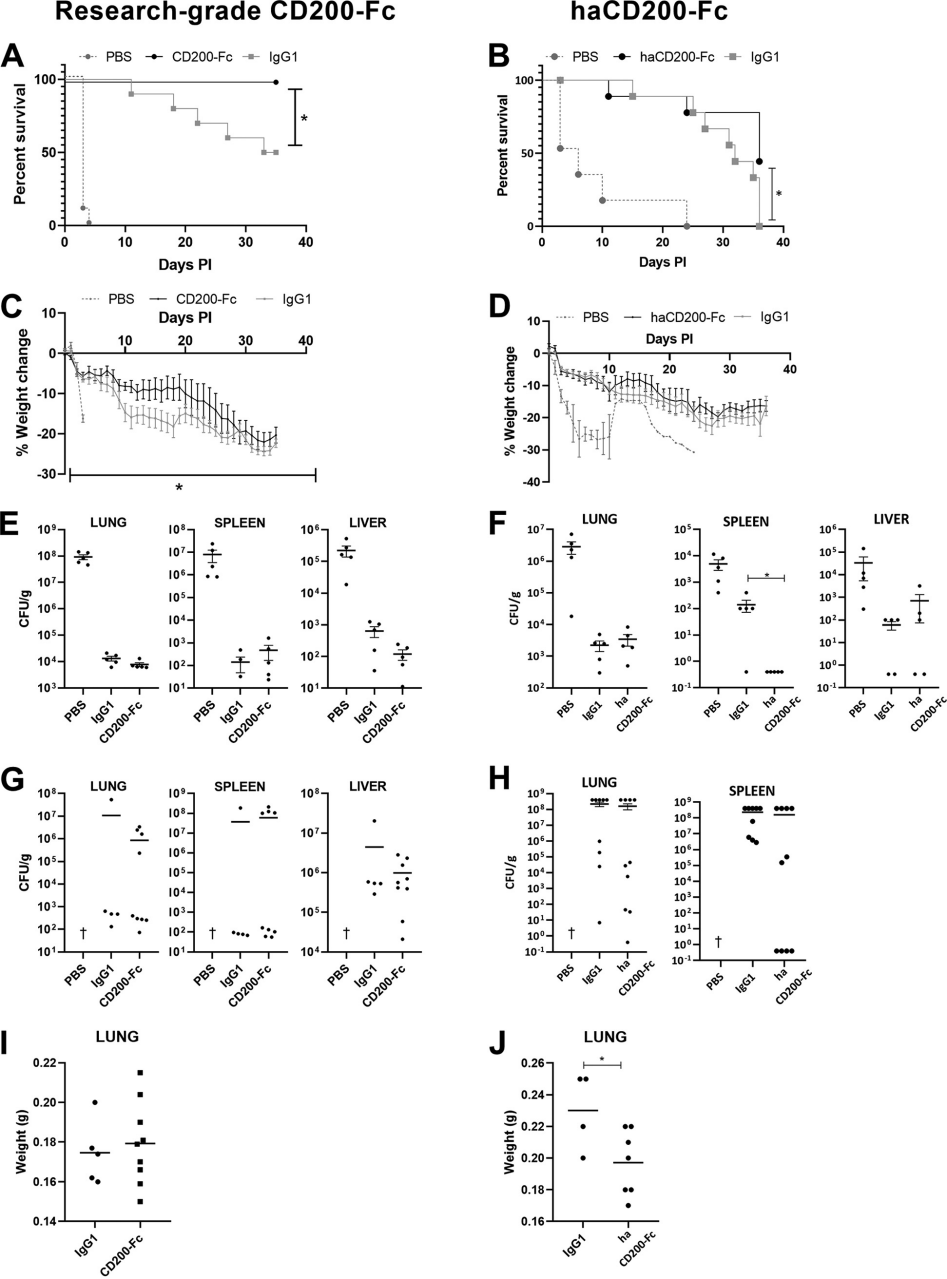
Combinational therapy to treat B. pseudomallei infection *in vivo.* BALB/c mice were challenged with 100 CFU (25 LD_50_) B. pseudomallei via the aerosol route and treated with doxycycline (orally) in combination with either research-grade (3 mg/kg) or haCD200-Fc (1.25 mg/kg) (left and right, respectively), isotype control antibody (intraperitoneal). For each treatment group, *n* = 10. An additional group of mice were challenged and treated with PBS only (*n* = 5). Each day following infection, the mice were scored for adverse clinical signs of disease weighted. (A and B), Survival of mice up to 40 days post-infection; data were analyzed using the log-rank (Mantel-Cox) test; *, *P* < 0.05. (C and D) Weight change calculated as a percent from day 0 post-infection; data were analyzed using two-way analysis of variance (ANOVA) with mixed effects multiple comparison. (E and F) Bacterial burden at day 3 post-infection within organs was determined and defined as CFU/gram of tissue (CFU/g) (*n* = 5 per treatment group). (G and H) Bacterial burden at day 35 post-infection within organs was determined and defined as CFU/gram of tissue (CFU/g) (*n* = 10 per treatment group). (I and J) Lung weights at day 35 post-infection (*n* = 10 per treatment group). (E to G) The data were analyzed using Student’s *t* test; *, *P* < 0.05. All data represent mean ± SEM. †, Animals reached humane endpoint prior to schedule cull.

Mice were aerosol challenged with 100 CFU (25 LD_50_) B. pseudomallei (K96243). At days 1, 3, and 7, all treatment groups received i.p. injections of either research-grade CD200-Fc, haCD200-Fc (1.25 mg/kg to 3 mg/kg) or relevant IgG1 control. For the first 7 days post-infection, all groups were orally administered daily with doxycycline monohydrate (Vibramycin; Pfizer) (100 mg/kg); the dose chosen is known to be a suboptimal therapeutic dose (18). A final group of mice received phosphate-buffered saline (PBS) via the oral and i.p. routes to control for treatment administered by both routes. Mice were weighed and scored for clinical signs of infection twice daily and euthanized if they reached criteria for the humane endpoint. All treatment groups resulted in a significant increase in survival compared with the PBS group (*P* < 0.0001) (Fig. 2A and B). Importantly, there was a statistically significant increase in the survival of mice treated with CD200-Fc and doxycycline compared with that of control mice (IgG and doxycycline), with both studies showing a 50% increase in survival (Fig. 2A and B). These data build on existing layered defense approaches for infectious disease (19–21) but are the first to report the use of an immunomodulator targeting a natural regulatory mechanism which is particularly active in the lung environment. Percentage body weight loss was calculated from day 0 post-infection, and data suggested that mice treated with CD200-Fc in combination with doxycycline lost less weight than antibiotic control-treated mice (Fig. 2C and D), indicating a healthier animal state.

Planned culls were performed at day 3 post-infection to determine bacterial burden in the organ tissues. There was a reduction in bacterial burden in all tissues following treatment of either CD200-Fc compared with that of the PBS control, but this reduction was not significant in the lung and liver due to the isotype effects of the control antibody (Fig. 2E and F). A significant difference was observed in the spleen tissue (*P* < 0.05) following treatment with haCD200-Fc compared with the isotype control (Fig. 2F). Interestingly, we have shown that when CD200-Fc is combined with a different class of antibiotic, co-trimoxazole, we do see a consistent reduction in liver infection at day 3 post-infection, which is statistically significant (see Fig. S1 in the supplemental material). However, the model requires further development to determine if there is a survival advantage. Bacterial burden in the organ tissues was also enumerated at the end of the study (Fig. 2G and H). All mice harbored bacteria, but due to the chronic nature of the disease, the bacterial loads were variable and we did not see any differences between antibiotic and dual treatment. The weight of each organ was measured at the 35 day post-infection post-mortem. There were no differences in organ weights seen in mice treated with the research-grade CD200-Fc (Fig. 2I). In the mice treated with the haCD200-Fc, significant differences were seen in the lung tissue compared with the control group (Fig. 2J). This finding may be due to the fact that the Ducentis compound has been engineered specifically for higher affinity binding to CD200R1.

Planned culls were also performed at day 8 post-infection; at this time point, the mice had received three doses of the immunomodulatory compound. A 23-plex immunological biomarker panel was used to measure the cytokines present in the lung, spleen, and liver. Significant differences were observed in mice treated with doxycycline in combination with CD200-Fc compared with those treated with IgG control (Table 1). Of the biomarkers up-regulated, both Th1 and Th2 cytokines were significantly induced following treatment with CD200-Fc. Of the Th1 cytokines, interferon gamma (IFN-γ), granulocyte-macrophage colony-stimulating factor (GM-CSF), and interleukin-12 (IL-12) are known to support cellular host responses and activate macrophage bactericidal activity. The upregulation of these Th1 cytokines through the CD200 receptor pathway is in line with previous data where we have shown an induction of reactive oxygen species through the same pathway (6). Furthermore, the induction of Th2 cytokines IL-4, IL-5, IL-9, and IL-10 is known to be associated with tissue repair and anti-inflammatory responses that have a role in protecting the host from tissue damage. The activation of both arms of the cellular host response could imply the induction of an immunological environment to allow for killing of the invading bacterial agent as well as protection of the host that could cause otherwise damaging levels of pro-inflammatory cytokines. At this time point, no differences in bacterial burden between groups were observed (data not shown).

**TABLE 1:** Summary of immunological biomarkers upregulated at day 8 post-infection following combinational therapeutic treatment^*a*^

Biomarker	Doxycycline + CD200-Fc v doxycycline + IgG1 treatment result by tissue		
	Lung	Spleen	Liver
Eotaxin	↑ *		
GM-CSF	↑ *	↑ *	↑ *
IFN-γ	↑ *		
IL-1β			↑ *
IL-3	↑ *	↑ *	↑ *
IL-4	↑ **	↑ **	
IL-5	↑ **		↑ **
IL-10	↑ *		↑ *
IL-12 p70	↑ **		↑ *
IL-17A			↑ *
MIP-1β	↑ *		

a Mice were challenged with *B. psuedomallei* and treated with doxycycline in combination with CD200-Fc or IgG isotype control. At day 8 post-infection, lung, spleen and liver tissues were collected and homogenized. Using a 23-plex murine Luminex array (Bio-Rad) immunological biomarker panel, the homogenates were screened to identify cytokines up- and down-regulated. ↑, indicates up-regulation. Statistical analysis was performed using unpaired Student’s *t* test; *, *P* < 0.05; **, *P* < 0.01 (*n* = 5).

To determine if the beneficial effects of the combination treatment following infection were due to altering blood concentrations of either the antibiotic or antibody therapy, a pharmacokinetics (PK) study was conducted. Naive mice were administered orally with either doxycycline and/or the haCD200-Fc compound as a single dose on day 0. Animals were euthanized from 0.5 to 192 h and blood was collected for analysis of the compounds. Antibiotic concentration in the plasma indicated that there were no major advantageous changes in antibiotic drug concentration (to a common terminal time point) when it was administered in conjunction with haCD200-Fc (Table 2). The antibiotic concentrations were also in line with previous murine PK studies conducted in-house (18). haCD200-Fc levels in the serum were measured via enzyme-linked immunosorbent assay (ELISA) throughout the time course, and in mice, haCD200-Fc has a half-life of approximately 62 h (Table 3). This finding is in line with other antibody-based therapies (22) and demonstrates that the beneficial effects were not caused by changes in antibiotic PK. Therefore, we hypothesize that improvements in outcome following infection were a result of the immunomodulatory capacity of the CD200-Fc compound.

**TABLE 2:** Doxycycline pharmokinetics^*a*^

Parameter	Unit	Data by treatment	
		CD200-Fc and doxycycline	Doxycycline only
Absorption (*k_a_*)	$h^{-1}$	>100	1.68
Elimination (*k_e_*)	$h^{-1}$	0.21	0.27
Time to maximum concn (*t_max_*)	h	<1	1.24
Maximum concn (*C_max_*)	ng · μL	2.04	2.52
Half-life (*t*_0.5_)	h	3.37	2.56
Area under the curve (AUC_48 h_)	mg · h . L^−1^	8.9	12.7

a Mice were orally administered a single dose of doxycycline (100 mg/kg) with and without i.p. injection of the haCD200-Fc compound (3 mg/kg) on day 0. Naive animals administered with PBS were used as controls. Animals were euthanized at 0.5, 1, 4, 8, 24, 48, 96, or 192 h and blood was collected for analysis of the compounds. *n* = 4 for each time point. Antibiotic concentration in the plasma was determined via liquid chromatograph-mass spectrometry (Agilent 1100; CTC PAL, Sciex API3000, Sciex API4000). PK parameters were calculated using a compartmentalized model.

**TABLE 3:** haCD200-Fc pharmokinetics^*a*^

Parameter	Units	haCD200-Fc
Vol of distribution (*Vd*)	μL	460
Absorption (*k_a_*)	$h^{-1}$	3.30
Elimination (*k_e_*)	$h^{-1}$	0.01
Time to maximum concn (*t_max_*)	h	1.73
Maximum concn (*C_max_*)	ng · μL	159.89
Half-life (*t*_0.5_)	h	62.29
Clearance (*CI*)	μL · h^−1^	5.12

a Mice were administered a single dose of haCD200-Fc compound (3 mg/kg) via the i.p. route on day 0. Naive animals administered with PBS were used as controls. Animals were euthanized at 0.5, 1, 4, 8, 24, 48, 96, or 192 h and blood was collected for analysis of the compounds. *n* = 4 for each time point. haCD200-Fc concentration in the serum was measured by ELISA that was undertaken by Syngene Ltd. PK parameters were calculated using a compartmentalized model.

Overall, our data suggest that an immunomodulator and antibiotic combination therapy approach is of benefit for the treatment of melioidosis and should be considered alongside other layered defense approaches. For example, it has been documented recently that the combination of a vaccine with an antibiotic (23) or the combination of two different antibiotics (18) are also superior to single-antibiotic-only treatments for B. pseudomallei infection.

### Animal ethics.

Animal studies were performed in accordance with the United Kingdom Scientific Procedures Act (Animals) 1986 and the United Kingdom Codes of Practice for the Housing and Care of Animals Used in Scientific Procedures, 1989. Investigations involving animals were carried out according to the requirements of the UK Animal (Scientific Procedures) Act 1986 under the authority of Project License P1D46FB69 granted by the UK Home Office and an Animal Care and Use Review Office (ACURO) Appendix. This project license was approved by the Animal Welfare and Ethical Review Body (AWERB), Dstl, Porton Down which includes a review panel consisting of Named Veterinary Surgeon (NVS), Named Animal Care and Welfare Officer (NACWO), Named Training and Competency Officer (NTCO), Named Information Officer (NIO), Home Office Liaison Contact (HOLC) and independent non-technical members.
