# Supplementary material for: Assessment of CD200R Activation in Combination with Doxycycline in a Model of Melioidosis
Source: Microbiol Spectr. 2023 May 18;11(3):e04016-22. doi: 10.1128/spectrum.04016-22 (PMC10269878; doi:10.1128/spectrum.04016-22)
Supplement: Supplemental file 1 — Supplemental material. Download spectrum.04016-22-s0001.pdf, PDF file, 0.7 MB [file spectrum.04016-22-s0001.pdf]

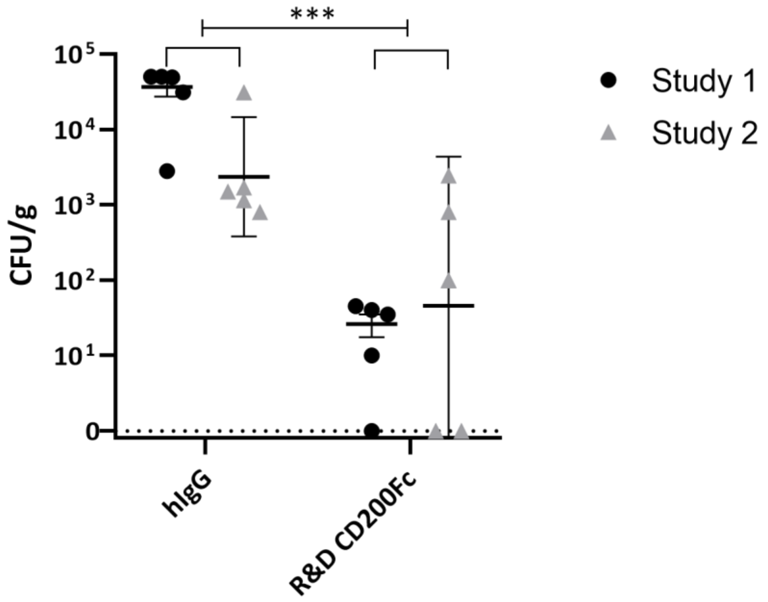

Supplementary Figure 1:

Bacterial burden following combinational therapeutic treatment.

Balb/C mice were challenged with 100 CFU *B. pseudomallei* via the aerosol route and treated with co-trimoxazole in combination with either IgG or CD200-Fc. At day 3 post-infection bacterial burden in the liver tissues were determined by plating out the tissue homogenates. Bacterial burden was defined as colony forming units/g (CFU/g). For each treatment group n=5 and the experiment was repeated on two separate occasions. Data were analyzed using two-way ANOVA, where *** $p < 0.001$.
